# The relevance of network micro-structure for neural dynamics

**DOI:** 10.3389/fncom.2013.00072

**Published:** 2013-06-04

**Authors:** Volker Pernice, Moritz Deger, Stefano Cardanobile, Stefan Rotter

**Affiliations:** Bernstein Center Freiburg and Faculty of Biology, Albert-Ludwig UniversityFreiburg, Germany

**Keywords:** cortical networks, microstructure, integrate-and-fire neuron, multifractal network generator, asynchronous irregular state

## Abstract

The activity of cortical neurons is determined by the input they receive from presynaptic neurons. Many previous studies have investigated how specific aspects of the statistics of the input affect the spike trains of single neurons and neurons in recurrent networks. However, typically very simple random network models are considered in such studies. Here we use a recently developed algorithm to construct networks based on a quasi-fractal probability measure which are much more variable than commonly used network models, and which therefore promise to sample the space of recurrent networks in a more exhaustive fashion than previously possible. We use the generated graphs as the underlying network topology in simulations of networks of integrate-and-fire neurons in an asynchronous and irregular state. Based on an extensive dataset of networks and neuronal simulations we assess statistical relations between features of the network structure and the spiking activity. Our results highlight the strong influence that some details of the network structure have on the activity dynamics of both single neurons and populations, even if some global network parameters are kept fixed. We observe specific and consistent relations between activity characteristics like spike-train irregularity or correlations and network properties, for example the distributions of the numbers of in- and outgoing connections or clustering. Exploiting these relations, we demonstrate that it is possible to estimate structural characteristics of the network from activity data. We also assess higher order correlations of spiking activity in the various networks considered here, and find that their occurrence strongly depends on the network structure. These results provide directions for further theoretical studies on recurrent networks, as well as new ways to interpret spike train recordings from neural circuits.

## 1. Introduction

The influence of single neuron properties as well as global network parameters on the population dynamics of neurons has been subject of a large number of studies (Brunel, 2000; Vogels et al., 2005; Kumar et al., 2008; Benayoun et al., 2010; Mongillo et al., 2012). Often, a simple paradigm to assign connections between neurons is assumed, like Erdös-Rényi random graphs, or all-to-all connectivity. Given such a network structure, additional parameters like the synaptic weights or the external input are varied, and effects on quantities like network oscillations, spike train irregularity or activity correlations are studied.

The influence of structural properties of neural networks on their dynamics has only recently begun to receive increased attention. One example is the study of neural dynamics at the scale of cortical regions (Bullmore and Sporns, 2009). On the level of individual neurons, the effects of a more realistic spatial arrangement have also been addressed in a number of studies. Realistic connection probabilities between layers were shown to reproduce measured rate distributions (Potjans and Diesmann, 2012). The correlations in large-scale networks with distance dependent connectivity were studied in Yger et al. (2011), and a large variety of spatio-temporal activity patterns were described in Voges and Perrinet (2009, 2012), where the spectrum of network topologies was also extended to networks with patchy connections.

However, on small scales, specific connectivity that cannot be inferred from the spatial positioning of the neurons alone is conceivable and has also been detected in experiments. The observed deviations from random structure include the abundance of specific network motifs (Song et al., 2005), distributed cell assemblies (Perin et al., 2011) or subnetworks of neurons with high firing rates (Yassin et al., 2010). These kinds of variations in the topology are usually described in the context of graph theory (Rubinov and Sporns, 2010). The dynamical implications of network structure on bursting activity have been explored in Gaiteri and Rubin (2011) and Mäki-Marttunen et al. (2011). The influence of motifs with two connections on the ability of excitatory networks to synchronize was analyzed in Zhao et al. (2011), and their effects on correlations in a linear framework in Hu et al. (2013). In Roxin (2011) it was shown that broadly distributed in- and out-degrees can promote oscillations in networks of integrate-and-fire neurons, and the effect of clustered connections was examined in Litwin-Kumar and Doiron (2012). Here, we want to study the effects of general variations in local connectivity in a recurrent network in an approximately asynchronous and irregular state (Brunel, 2000). This paradigm is motivated by the experimental finding of small correlations and irregular activity in many areas of the brain (Softky and Koch, 1993; Ecker et al., 2010; Cohen and Kohn, 2011; Barth and Poulet, 2012) and is commonly used in theoretical studies on the dynamics of recurrent networks of spiking neurons (Kumar et al., 2008; Renart et al., 2010; Yger et al., 2011) as a model of cortical activity.

One approach to analyze the effect of a particular network characteristic is to choose a specific parameterized network model, where this characteristic can be varied, to single out its effect on the dynamics. A common problem is to find a model that leaves the remaining properties of the network unchanged. As we have shown in Cardanobile et al. (2012) it is often the case that, by the construction principle of a specific network model, artificial dependencies between various network properties arise, such that, as a result, they cannot be varied independently. This compromises the generality of the results obtained with such models. Additionally, it is unclear if a certain network model captures all features observed in real networks. For example hubs, clustering or communities in networks all might have cooperating or competing effects on a certain dynamic property.

In this study, an alternative approach was applied. Rather than varying a specific network property, a large number of different networks was generated, their properties asserted (as measured by common statistical measures used in graph theory) and relations to dynamical properties established by means of numerical simulations. The rationale is that in this way the interplay of various network properties can be examined without the need to construct a specific model for each of them. A tool that is able to generate networks which vary with respect to a large number of common graph-theoretical measures is the multifractal network generator described in Palla (2010). A relatively dense connectivity (we use an average connection probability of 0.1) as well as directed connections can easily be realized, making the model suitable for the study of neural networks. The generated network ensemble is very variable with respect to many of the statistics commonly used to describe the properties of a neocortical network, like degree distributions and correlations, clustering, modularity and motif distributions. Also, only a small number of links between different network properties are introduced (Cardanobile et al., 2012). As networks are constructed on the level of the connectivity matrix, the nodes are not embedded in a metric space and no distance measure is applicable. These networks are thought to represent a local network of neurons residing in a about a cubic millimeter of the cortex. In such networks neurons might potentially form connections to any other neuron (Kalisman et al., 2005).

To assess the effects on the network dynamics, the generated networks were employed as the synaptic connectivity matrix of a neural network in a specific activity regime. To select this activity regime, parameters were adapted to generate asynchronous-irregular activity in a network of leaky integrate-and-fire neurons (LIF) in a random network (Brunel, 2000). This approach is necessarily restricted to a specific setting of both neuron and global network parameters and has, therefore, the character of a case study in this dimension. However, it enables the identification of the set of features or combinations of features that affect the network dynamics most strongly without the strong restrictions on structure implicitly used by simple network models. Because the full connectivity matrix of large neural networks is currently not accessible to experiments, such a general approach is necessary to evaluate the importance of network structure and will be helpful to determine relevant quantities as well as to interpret their values, once they become available.

## 2. Methods

### 2.1. Network generation

**Figure 1:**
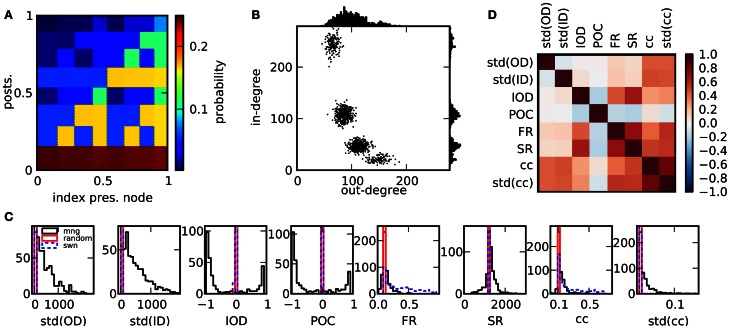
**Properties of the network ensemble. (A)** Sample for a probability function that can be used to generate networks. **(B)** Scatter plot of out-degrees vs. in-degrees of single nodes for a network realization (1000 nodes) resulting from the probability measure in **(A)** as well as degree distributions. **(C)** Comparison of histograms of selected features over 500 network realizations for different network models. The ensemble realized by the multifractal network generator (mng) shows a much larger variability in comparison to random networks or small world networks in most features. **(D)** Matrix of Pearson correlation coefficients among graph features across network realizations.

We created a total number of 2500 networks with 12,500 nodes each, using the multifractal network generator for the generation of the networks (Palla, 2010). In short, connections between nodes are established on the basis of a rugged probability function *p*(*x, x*′) defined over the interval [0, 1] × [0, 1]. Each node *i* is assigned a random continuous index *x*_*i*_ ∈ [0, 1]. A directed connection between two nodes is realized with probability *p*(*x*_*i*_, *x*_*j*_). The probability function is constructed in the following way: A function *p*_1_ is initiated by dividing [0, 1] × [0, 1] into a number of *m*^2^ rectangles, using *m* divisions on each the x- and the y-axes (constructed from *m* − 1 random uniformly drawn boundaries ∈ (0, 1)). The value of *p*_1_ in each rectangle is a constant randomly chosen from [0, 1]. In the next step, *p*_2_ is determined, by replacing each rectangle with an appropriately scaled version of the initial measure *p*_1_, multiplied by the constant value of *p*_1_ within this rectangle. This procedure is iterated *k* times. In each step, each rectangle of the current measure is multiplied by the initial measure *p*_1_. We used *m* = 2 and *k* = 4 and normalized the resulting *p*_*k*_ to an average value of 0.1. Due to the randomized initial conditions and the ensuing procedure, each network results from a radically different probability measure which determines its statistical properties, see Figure 1A for a specific realization. One way to characterize a network is by the distribution of in- and outgoing connections (in- and out-degrees) across nodes, Figure 1B. Here, the expected out-degree of a node with index *x*_*i*_ is proportional to the integral of the probability measure over the vertical direction, ∫ *p*(*x*_*i*_, *y*)*dy*. Consequently, along with the probability measure, degree distributions, correlations between degrees and other statistics are highly variable across networks. A constant measure *p*_1_ results in a random network of Erdös-Rényi type. Note that in this study we do not impose a symmetry condition on *p*_1_ and generate directed networks, in contrast to Palla (2010).

### 2.2. Network simulations

We simulated networks of current-based LIF neurons in an asynchronous irregular state, with parameters similar to the ones in Brunel (2000). The membrane potential *V*_*i*_ of neuron *i* evolves according to the differential equation

$$\tau_{m}\frac{dV_{i}}{dt}=-V_{i}+\sum_{j}J_{ij}s_{j}(t-d)+J_{\text{ext}}s_{\text{ext}}(t).$$

The time constant of the membrane potential is denoted by τ_*m*_, synaptic weights *J*_*ij*_ were set depending on the presynaptic neuron type and the connectivity matrix constructed as described in section 2.1. If the membrane potential exceeded a threshold *V*_th_, a spike was emitted and the membrane potential was set to a reset potential *V*_*r*_, where it was kept fixed for a refractory time *t*_*r*_. Synaptic currents were described as delta-functions, such that the input currents resulting from presynaptic spike trains were given by *s*_*j*_(*t*) = ∑_τ_δ(*t* − *t*^*j*^_τ_). For all connections, a constant synaptic delay *d* was used.

Supra-threshold external input to each neuron was provided by a spike train *s*_ext_(*t*) with a coupling weight *J*_ext_ and spike times modeled as a Poisson process with rate ν_ext_ = 2*V*_th_/(*J*_ext_τ_*m*_). A randomly chosen fraction of 20% of the neurons were inhibitory. As the synaptic weights of these neurons were stronger by a factor −5, networks were inhibition dominated. In combination with strong external input this evokes stable asynchronous and irregular firing in random networks. Simulations were conducted using the NEST simulator (Gewaltig and Diesmann, 2007). The first second of the simulated time was discarded, so that the network activity could reach a steady state. Numerical values of all simulation parameters are summarized in Table 1.

**Table 1:** **Simulation parameters**.

**Parameter**	**Symbol**	**Value**
Number of neurons	*N*	12,500
Number excitatory neurons	*N*_*E*_	10,000
Number inhibitory neurons	*N*_*I*_	2500
Average connection probability	$\bar{p}$	0.1
Excitatory weight	*J*_*E*_	0.1 mV
Inhibitory weight	*J*_*I*_	−0.5 mV
Ratio excitation to inhibition	*g*	5
Membrane time constant	τ_*m*_	20 ms
Refractory time	τ_*r*_	2 ms
Synaptic delay	*d*	1.5 ms
Threshold potential	*V*_th_	20 mV
Reset potential	*V*_*r*_	10 mV
Equilibrium voltage	*E*_*L*_	0 mV
Weight of external inputs	*J*_ext_	0.1 mV
Rate of external inputs	ν_ext_	20 kHz
Simulation time	*t*_sim_	11 s
Small bin size for spike counts	Δ_*s*_	5 ms
Large bin size for spike counts	Δ_*l*_	100 ms
Frequency range power spectrum	*f*_min_, *f*_max_	0.1 Hz, 1000 Hz

### 2.3. Measures of features

We use a variety of features commonly applied in the literature to characterize both the structure of the network and the activity of the simulated spike trains. They are summarized in Tables 2, 3. For a quantity *a*, we denote by std(*a*[*i*])_*i*_ the empirical standard deviation (over nodes or time bins *i*), by corr(*a*[*i*], *b*[*i*])_*i*_ the Pearson correlation coefficient, and by 〈*a*[*i*]〉_*i*_ the mean across the index *i*. The Fourier transform of a function *f*[*t*] is denoted by FT(*f*[*t*]). Only scalar values were chosen as features. Mean, standard deviation and correlations of features across networks will be examined in sections 3.1 to 3.3. The variation of characteristics of individual nodes within a single network realization will be addressed in section 3.4.

**Table 2:** **Network features**.

**Description**	**Definition**	**Abbreviation**
Adjacency matrix	*A*_*ij*_	
In-degree	*d*^+^(*i*) = ∑_*j*_*A*_*ij*_	
Out-degree	*d*^−^(*j*) = ∑_*i*_*A*_*ij*_	
Standard deviation of in-degrees	std(d^+^[i])_i_	std(ID)
Standard deviation of out-degrees	std(d^−^[j])_j_	std(OD)
Correlation between in- and out-degree	corr (*d*^+^[*i*], *d*^−^[*i*])_*i*_	IOD
Directed clustering coefficient	$\text{cc}(i)=\frac{{\left[{\left(A+A^{T}\right)}^{3}\right]}_{ii}}{2([d^{+}+d^{-}][d^{+}+d^{-}-1])-2{\left[A^{2}\right]}_{ii}}$	
Average clustering coefficient	〈cc[*i*]〉_*i*_	cc
Standard deviation of clustering coefficients	std(cc[i])_i_	std(cc)
Spectral radius of *A*	ρ(*A*)	SR
Fraction of recurrent connections	$\frac{\sum_{ij}A_{ij}A_{ji}}{\sum_{ij}A_{ij}}$	FR
Postsynaptic neurons of *i*	*i*′	
Correlation out-degree vs. postsynaptic out-degree	corr (*d*^−^[*i*], 〈*d*^−^[*i*']〉_*i*'_)_*i*_	POC
Number of excitatory, inhibitory inputs	*d*^+^_*I*_[*i*], *d*^+^_*E*_[*i*]	
Local excitation-inhibition ratio	${\text{g}}_{\text{loc}}(i)=g\frac{d_{I}^{+}[i]N_{E}}{d_{E}^{+}[i]N_{I}}$	

**Table 3:** **Activity features**.

**Description**	**Definition**	**Abbreviation**
Spike times of neuron *i*	*t*^*i*^_τ_	
Spike train of neuron *i*	*s*_*i*_(*t*) = ∑_τ_δ(*t* − *t*^*i*^_τ_)	
Number of spikes of *i* during the simulation	*n*_*i*_	
Inter spike intervals	ISI^*i*^_τ_ = *t*^*i*^_τ+1_ − *t*^*i*^_τ_	
Coefficient of variation	$\text{CV}[i]=\frac{\text{std}{\left({\text{ISI}}_{\tau }^{\text{i}}\right)}_{\tau }}{{\langle {\text{ISI}}_{\tau }^{i}\rangle }_{\tau }}$	
Mean CV	〈CV[i]〉_*i*_	CV
Standard deviation of CV	std(CV[i])_*i*_	std(CV)
Spike count of neuron *i* in bin number τ of size Δ	*c*^*i*^_τ_(Δ)	
Highest order of detectable higher-order correlations	See text	HOC
Mean count correlation for small/large bin size	〈corr(*c*^*i*^_τ_[Δ_*s,l*_], *c*^*j*^_τ_[Δ_*s,l*_])_τ_〉_*i≠j*_	CCC_*s/l*_
Standard deviation of count correlation	std(corr(*c*^*i*^_τ_[Δ_*s/l*_], *c*^*j*^_τ_[Δ_*s/l*_])_τ_)_*i≠j*_	std(CCC_s/l_)
Mean spike rate	${\langle \frac{n_{i}}{t_{\text{sim}}}\rangle }_{i}$	rate
Standard deviation of the rate	$\text{std}{\left(\frac{{\text{n}}_{\text{i}}}{{\text{t}}_{\text{sim}}}\right)}_{\text{i}}$	std(rate)
Mean of rates, neurons with rate >0	${\langle \frac{n_{i}}{t_{\text{sim}}}\rangle }_{i:n_{i}>0}$	rate^+^
Standard deviation rate, neurons with rate >0	$\text{std}{\left(\frac{{\text{n}}_{\text{i}}}{{\text{t}}_{\text{sim}}}\right)}_{i:n_{i}>0}$	std(rate^+^)
Population activity	*S*(*t*) = ∑_*i*_*s*_*i*_(*t*)	
Normalized power spectrum	*P*(ω) = (|FT[*S*(*t*)]|^2^ − 〈*S*(*t*)〉_*t*_)/(〈*S*(*t*)〉^2^_*t*_ *t*_sim_)	
Integrated spectral power of population activity	$\int_{f_{\text{min}}}^{f_{\text{max}}}P(\omega )d\omega$	Spec

A variety of quantities has been found to influence the dynamics of networks (Boccaletti et al., 2006). In many applications, the statistics of node degrees (numbers of incoming and outgoing connections) are of interest, as real networks often exhibit heavy-tailed degree distributions (Barabási and Albert, 1999). As the mean degree in our networks is fixed, we use the standard deviation of in-degree and out-degree distributions as graph features. To assess degree correlations, we furthermore consider the correlation between the out-degree of a node with the out-degrees of its postsynaptic neurons, as well as the correlation between the in- and the out-degree. These are two independent measures of degree correlations in directed networks accounting for slightly different properties than for example the assortativity index proposed in Newman (2002). Since the introduction of the small world network model (Watts and Strogatz, 1998), the clustering coefficient has been an equally prevalent measure. To evaluate the influence of clustering, we use the directed clustering coefficient (Fagiolo, 2007), as well as the fraction of realized bidirectional connections in networks. Also, spectral properties of a network have often been used as an indicator for the dynamics of a system, for example to assess the tendency to synchronize (Atay et al., 2006). Here, we use the spectral radius, that is the modulus of the largest eigenvalue of the connectivity matrix, as a feature. To classify the input of an individual neuron, we will use the local ratio of its excitatory and inhibitory input.

In order to measure properties of the network activity, we use the following descriptors: the simplest features concern the mean and variance of the observed firing rates. As a number of neurons possibly receive a surplus of inhibitory input and remain silent, we also included mean and variance of the active, observable population. To capture the ubiquitous irregularity in spike trains, we use the coefficient of variation of the individual neurons' inter-spike intervals. For a quantification of the amount of synchrony, we measure spike count correlations in pairs of neurons, both for short and for long bin sizes. Alternatively, the oscillations in the population activity can be captured by the power spectrum of the combined spike trains. A comparable measure for network realizations with different firing rates was obtained by subtracting the constant offset of the spectrum and normalizing the peak at frequency zero to 1. We used the integral over the normalized power spectrum as a scalar measure (see Table 3 for details). Finally, we measure the maximum degree of higher-order correlations that can be detected in the observed activity using an algorithm proposed in Staude et al. (2010b), with parameters α = 5% (significance level) and *m*_max_ = 4 (cumulants of the population activity up to order 3 are used). In short, it is tested whether groups of neurons have a tendency to spike together beyond what can be expected from the correlations among smaller sub-groups. The largest group size that can significantly be inferred in the given dataset is returned.

In Figure 1C the variability of the network features in the chosen network ensemble is demonstrated by comparison of the resulting feature distributions with the ones from alternative network models. The feature distributions of both random and small world networks are generally much narrower, indicating that different network realizations within these ensembles are much more alike in their statistical properties. Only specific network features, like the clustering coefficient are varied substantially. These other networks had the same number of nodes and the same expected number of connections: in random networks, each connection was established with a probability *p* = 0.1. Small world networks were based on a ring structure where each neuron was connected to its *pN* nearest neighbors. Subsequently, outgoing connections were rewired with a constant probability uniformly chosen from the interval [0, 1] for each network, thus interpolating between fully random and ring networks. We quantify statistical relations between features with the empirical Pearson correlation coefficient across network realizations. In panel **(D)**, the correlations between structural features are depicted. Several network properties are correlated for the ensemble of networks under consideration. This can be due to either mathematical relations among the corresponding quantities, which are valid for graphs in general, or to the specific algorithm used here to generate networks. Nonetheless, as described in Cardanobile et al. (2012), the features in the ensemble accessible to the fractal network generator exhibit both a greater variability and a greater independence from each other in their variation than in the more traditional network models.

The correlation coefficients provide a good basis to assess mathematical relations between features. Strictly speaking, the correlation coefficient is a measure for the strength of a linear relationship between variables. Because of that, it is possibly not sufficient to detect interesting non-linear relations. To rule out non-linear dependencies, we also calculated the maximum information coefficient (MIC) using the toolbox MINE (Reshef et al., 2011). It turned out that no new relations were discovered by this alternative measure, which was introduced as a means to detect non-linear relations in large sets of variables (data not shown).

## 3. Results

### 3.1. Variation of statistics

The variation of the selected descriptors due to the variation of network structure is summarized in Table 4. The numbers were extracted from the ensemble of networks implicitly defined by the fractal network generator. These distributions should not be confused with the distributions of node and link properties of individual networks that are the basis for some network features. The variability of most of the structural features (bottom of the table), as measured by their coefficient of variation across networks, is reasonably large, underlining once more the broad range of different networks that can be generated. Many standard algorithms used to synthesize networks do not generate such variable ensembles (Cardanobile et al., 2012).

**Table 4:** **Variability of features across networks**.

**Feature**	**Mean**	**Std. dev.**	**CV = Std. dev./Mean**
CV	0.41	0.074	0.18
std(CV)	0.2	0.023	0.12
CCC_*s*_	0.018	0.011	0.61
std(CCC_*s*_)	0.028	0.0029	0.1
CCC_*l*_	0.0074	0.0042	0.57
std(CCC_l_)	0.1	0.012	0.12
rate	41	6.5	0.16
std(rate)	30	5.2	0.17
rate^+^	47	10	0.22
std(rate)^+^	27	3.1	0.11
Spec	0.015	0.011	0.73
HOC	81	81	1.00
std(OD)	480	400	0.84
std(ID)	470	410	0.87
IOD	0.12	0.86	7.5
POC	0.072	0.85	12.0
FR	0.13	0.072	0.54
§R	1300	250	0.2
cc	0.13	0.058	0.44
std(cc)	0.015	0.023	1.5

**Figure 2:**
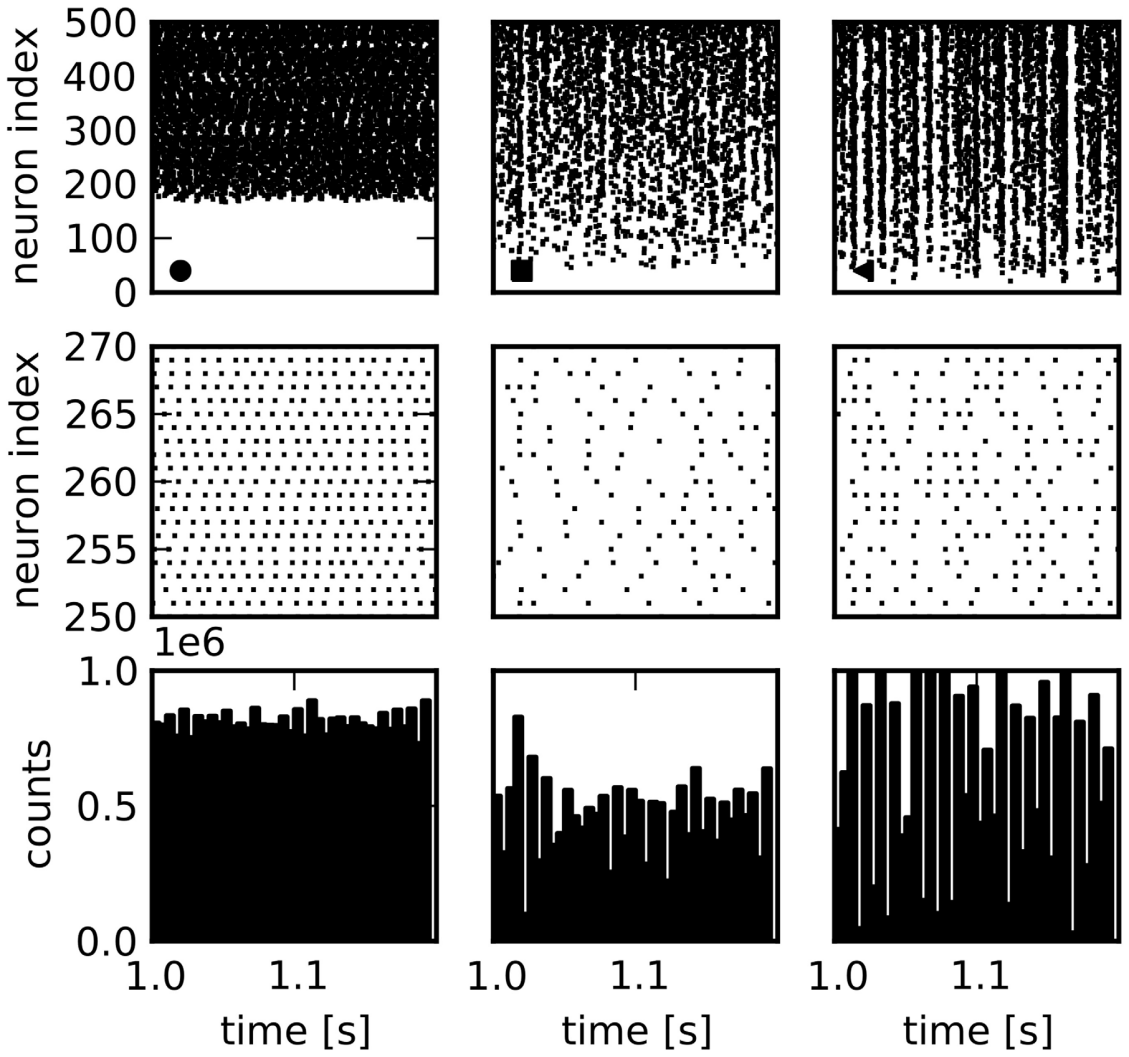
**Spike train raster plots for three network examples. Columns:** Activity in three different sample networks. **Top row:** Raster plots of activity of 500 randomly chosen neurons, sorted according to their firing rate. **Middle row:** Enlarged view of a smaller number of spike trains. **Bottom row:** Population activity of all neurons in bins of 5 ms demonstrates differences in total population activity and fluctuations.

**Figure 3:**
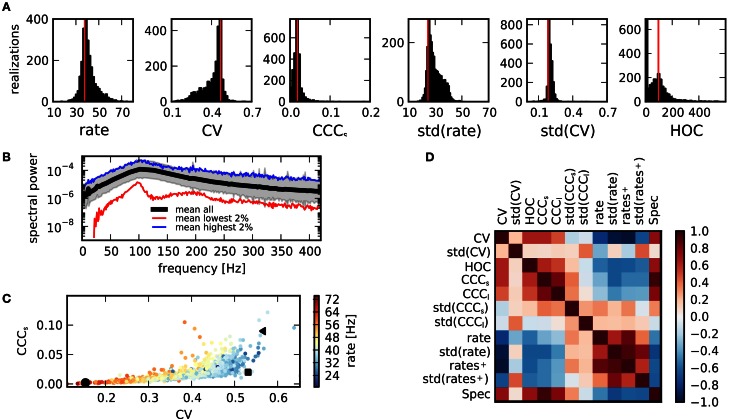
**Activity measures. (A)** Distribution of key statistical measures for activity across networks demonstrates the variety of realized activity patterns. Vertical red lines indicate corresponding values for a sample random network. **(B)** Power spectrum of population activity is similar across realizations. Black: Average across all networks. Shaded region shows sample average ± relative error calculated from standard deviation. Blue: Average across 2% of all networks with highest integrated power spectrum. Red: Average across 2% of networks with lowest integrated power spectrum. **(C)** Scatter plot of CV as a measure of spike train irregularity and CCC_*s*_, which quantifies the average pairwise correlation, for all networks. Colors indicate mean rate. Black symbols correspond with examples shown in Figure 1. The variations of these features are strongly correlated. **(D)** The matrix of correlations between all activity characteristics, computed for the sample of networks considered in this study, reveals the various dependencies between statistical measures.

The variability is generally somewhat lower for the features describing network activity. The median of the CVs for the distributions of structural features is 0.85, cf. Table 4, while it is 0.18 for the activity features. Nonetheless, very different activity regimes are realized. Figure 2 shows examples for the activity of three selected network realizations (cf. Figure 3C). While an asynchronous and irregular state is realized in some networks (middle column), there are also networks with strongly correlated spike trains (right) or very regular activity with high firing rates (left). Accordingly, the population activity differs strongly, both in mean and variance (bottom). The distributions of activity features vary in shape, see Figure 3A for examples. Nonetheless, their mean and variance can provide a good indication for their variation across networks. Remarkably, due to the synchronous activity in some networks, higher-order correlations of very high degrees can be detected. Hence, relatively large populations of neurons show indications of collective spiking. As for the pairwise spike count correlations, this depends strongly on the network realization. Our result suggests, however, that correlations of high order are a standard feature in recurrent networks, provided a sufficiently large population of neurons is observed.

In summary, even for identical global input, the activity exhibited by a network depends strongly on its structure. Yet, not all activity features are equally affected, as is indicated by the spectrum of the population activity, Figure 3B. While the absolute power, measured by the integrated power spectral density, varies across orders of magnitude, the shape, characterized by two peaks, remains similar for networks of high and low spectral power.

### 3.2. Relations between activity statistics

The values of features describing network activity are not independent. The scatter plot of CV (spike train irregularity) vs. CCC_*s*_ (correlations on a short timescale) in Figure 3C shows that in all networks with regular spike trains (low CV), correlations are also small. This means that a state of full synchrony (synchronous regular state) with low CV and high correlations is not realized. In contrast, in networks with large CV, both high and low correlations can be observed in different networks, suggesting that there is more variety in irregular states due to a more variable structure. As firing rates, indicated in color, tend to be higher in more regular states, it is apparent that the decrease of correlations or, equivalently, population fluctuations, for regular activity is not due to a general decrease of activity. Rather, irregular activity is only realized in networks with relatively low average rate.

The various dependencies between different activity features are summarized by a matrix of cross-correlation coefficients, computed across all networks in our ensemble, Figure 3D. Some of these relations are easily explainable. For example, the spectral power Spec is closely related to the strength of count correlations CCC_*s,l*_. Other relations are much less obvious: the widths of the distributions of correlation coefficients and CVs, std(CCC) and std(CV), appear to be less dependent on the values of other features. The mean values of rates, of the CV (of the interspike interval distribution), and correlations are, however, tightly interlinked: CCC_*s,l*_ (as well as other measures for correlations) is negatively correlated to the mean rate, but positively to the CV (see also panel **C**). The CV on the other hand, is negatively correlated with both the rate and its standard deviation. However, if only active neurons are considered, the negative correlation is much stronger with rate^+^ than with std(rate^+^). Some insight on this complex system of mutual dependencies can be gained by an inspection of the relations to the underlying properties of the network structure.

### 3.3. Relations between network and activity

**Figure 4:**
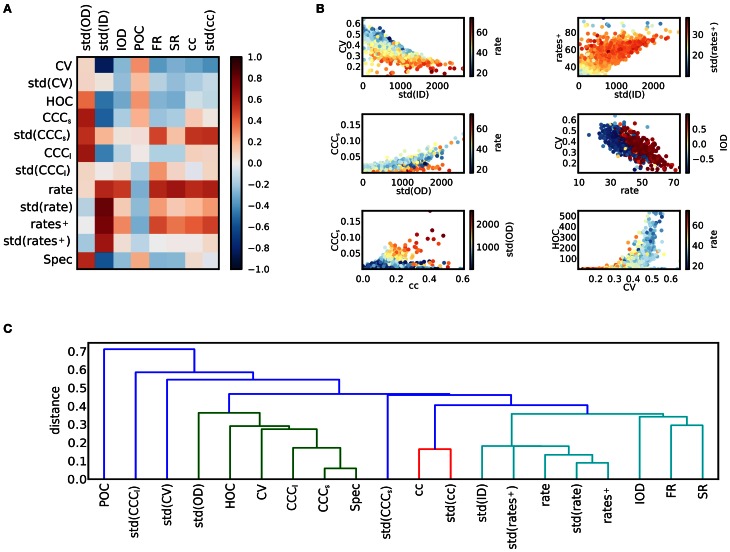
**Relations between activity and network structure. (A)** The matrix of correlation coefficients summarizes the effects of variations in network structure on spike train activity. **(B)** Scatter plots of selected pairs of features provide further information about the dependencies of various features (see main text). Colors indicate value of a third feature. **(C)** Dendrogram obtained after hierarchical clustering of the features. The distance measure is based on the correlation matrix. The level of the u-link indicates the distance between its children clusters (Jones et al., 2001). For illustration purposes, clusters that merge at a distance of 0.4 or larger are plotted in different colors. Clusters can be interpreted as families of features that are closely related.

The matrix of correlation coefficients between activity features and structural features is displayed in Figure 4A. In panel **(B)**, scatter plots of some features are singled out. As has been noted previously (Roxin, 2011), the width of the degree distributions strongly affects the activity. It appears that a high standard deviation for the in-degree implies low coefficients of variation. To understand this effect, the relation between std(ID) and the firing rates can be consulted: Strong correlations between std(ID) and the mean across all firing rates, and across rates of active neurons only, as well as std(rate) can be observed. A high variance in the in-degrees causes a large fraction of silent neurons with a high degree of inhibitory input as well as a fraction of neurons with correspondingly higher firing rates. Consequently, the variance of the firing rates is large. If only the active fraction is considered, the mean firing rate is more, and the rate variance less strongly correlated to std(ID), suggesting that high firing rates induce low coefficients of variations in a network. However, there are also other factors. It can be observed that for a given rate, the CV is higher, if a positive correlation between in-degrees and out-degrees in the network exists. To study the origin of these effects more closely, the relation between rate and CV of single neurons is discussed in section 3.4 below. The standard deviation of the out-degree std(OD) is strongly related to the strength of count correlations. This is not surprising, as a large out-degree variance corresponds to a large number of divergent motifs (Zhao et al., 2011; Hu et al., 2013). The relation appears to be stronger for networks with low mean rate. Additionally, std(OD) is related to the presence of higher-order correlations measured by HOC. The measures for degree correlations, POC and IOD, by themselves are apparently less important as a predictor for activity. Positive in-out-degree correlations strongly affect the mean rate only. This is very similar to the impact of the spectral radius of the network. Both quantities can to some degree be interpreted as measures how well activity in the network is propagated.

The clustering coefficient of a network also has implications for the network dynamics. It is only weakly related to the strength of correlations. In fact, high clustering coefficients can be due to a high out-degree variance, but also to a high in-degree variance (see Figure 1), and only high values of std(OD) induce high correlations. However, both the mean and the variance of the distribution of clustering coefficients are strongly related to count correlations on small timescales, CCC_*s*_, but not on large timescales, as the correlation to CCC_*l*_ is weak. Note, however, that CCC_*s*_ and CCC_*l*_ are correlated, cf. Figure 3. Finally, correlations of high order occur in networks of irregularly spiking neurons with rather low rates.

To provide an overview on the web of dependencies among the different features, the result of a hierarchical clustering algorithm on the matrix of feature correlations is shown in panel Figure 4C. As the distance measure between features *a* and *b*, we used 1 − corr(*a, b*), which is $\frac{1}{2}$ the squared Euclidean distance of the normalized variables, derived from the covariance as a bilinear product. It lies between 0 and 2 for perfectly correlated and perfectly anti-correlated variables, respectively. The quantitative results depend on the clustering algorithm as well as on the measure that is used to define the distance between clusters (here, the minimum distance between pairs of features belonging to each cluster was used), but the diagram illustrates a typical classification. For example, a group of features related to high firing rates can be distinguished, including the in-degree variance, the spectral radius and various measures related to clustering (light blue and red cluster). On the other hand, measures of correlations, the out-degree variance and the CV make up a second group (green cluster).

### 3.4. Features within networks

So far, we have only considered population statistics, as the mean and standard deviation across all nodes in a given network. In individual network realizations, also the statistics of the single neurons can be analyzed in order to elucidate the mechanisms leading to the activity of the full network. Here, we consider the relationship between rate and CV.

**Figure 5:**
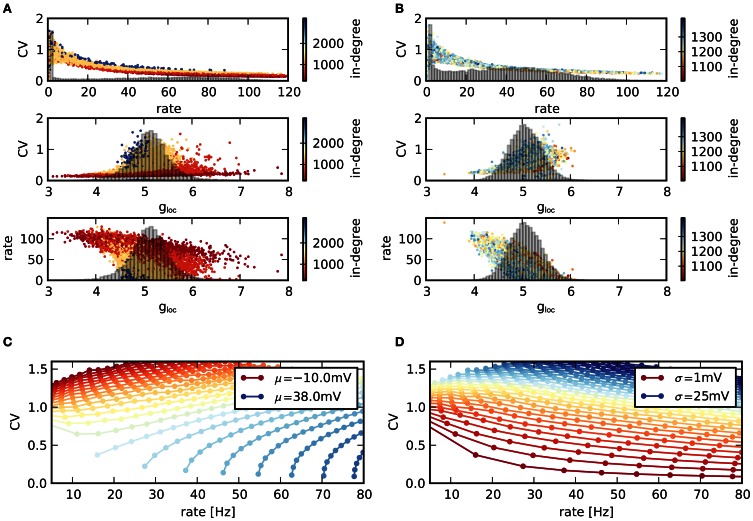
**Statistics of individual nodes. (A)** (Top) Scatter plot of rates vs. CVs across neurons in a single network with low average CV. Colors indicate the value of the in-degree of neurons. Histogram: distribution of rates in the network. (Middle) Corresponding scatter plot of CVs vs. excitation/inhibition ratio g_loc_ of the single neurons as well as distribution of g_loc_. (Bottom) Scattered rates vs. g_loc_. **(B)** Same as **(A)**, but for network with high average CV. **(C,D)** Relation between mean firing rate and CV of a single LIF neuron, depending on mean μ and standard deviation σ of voltage fluctuations. The parameters μ, σ denote the mean and standard deviation of the free membrane potential due to input currents. **(C)** Contours for constant μ and increasing σ (higher rates for higher σ). **(D)** Same data, but contours drawn for constant σ and increasing μ (higher rates for higher μ). The shape of the CV-rate dependency in neurons within networks [top panels in **(A,B)**] suggests that high firing rates result from an increase in input mean rather than variance.

As networks are very heterogeneous, the activity statistics of single neurons vary strongly, Figure 5. The two networks in panels **(A,B)** represent typical examples with low and high average CV. More precisely, networks ranked number 450 and 2100 according to their CV are displayed. In both networks, the CVs of individual neurons decrease with increasing rate, mirroring the relationship between CV and rate on the network level. The CV-rate curves are very similar in both cases. Hence, differences in mean CV can be related to the rate distribution within the network: a large number of neurons with high rates, and therefore low CVs lead to a low average CV (top panels in **A** and **B**). Note that silent neurons do not contribute to the average CV. Neurons differ in their activity due to differences in the input they receive. A larger ratio of inhibition g_loc_ in their population of presynaptic neurons causes both a larger CV and a smaller rate. Equally, for a given ratio of inhibition, a larger in-degree increases the CV, but decreases the rate (middle and bottom panels in Figures 5A,B).

This behavior can be related to the behavior of isolated LIF neurons receiving noisy input current. For g_loc_ > 4, the input is effectively inhibitory. As a result, a larger in-degree reduces the mean input and hence the rate. However, due to the larger number of afferents, the input variance increases, leading to more irregular spikes and a higher CV. The relationship between CV and rate is not unambiguous in LIF neurons, but depends on both mean and variance of the input current. In Figure 5C the simulated rate of a single LIF neuron is plotted against its CV, while the properties of the input current are varied. Here, μ, σ denote the expected membrane potential fluctuations that would result in a LIF neuron without spike threshold with corresponding current input. If the mean input current is held fixed and only its variance is changed, the CV increases with increasing rate. In contrast, if the variance is held fixed and the mean is varied, the CV decreases with increasing rate, panel **(D)**. Consequently, higher rates (resulting in lower CVs) are due to a larger mean input instead of a higher input variance in the recurrent network, as only a rate increase due to a higher mean input reproduces the observed relationship between firing rate and CV.

### 3.5. Predicting structure from activity

**Figure 6:**
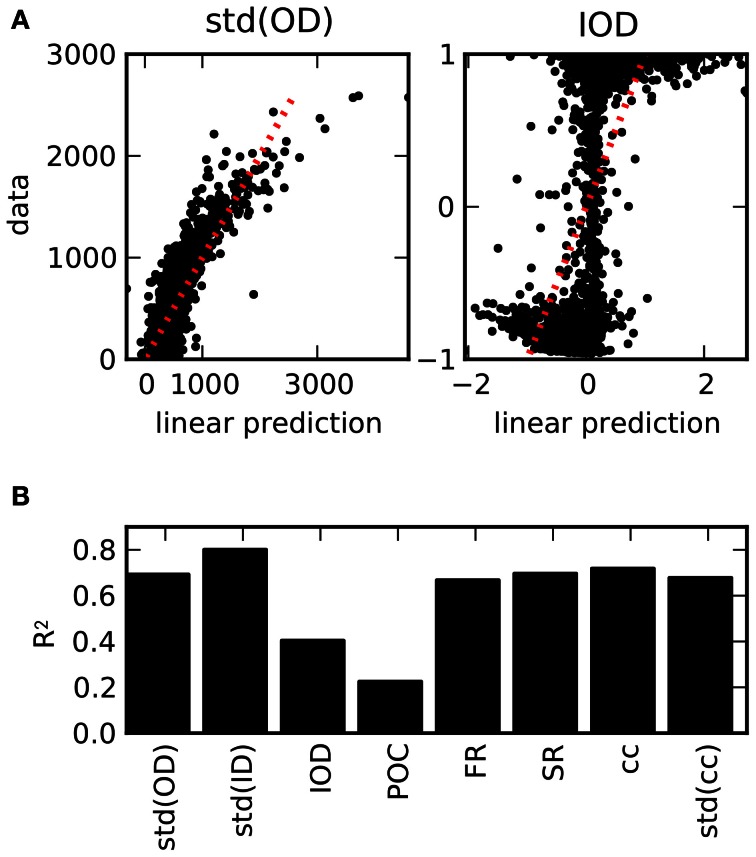
**Prediction of structural features on the basis of activity measures. (A)** Examples for the result of a linear regression across all network realizations on the basis of all 12 activity measures considered here (see Table 3). Scatter plot of the predictions from the linear regression against the exact values obtained from the connectivity matrix. **(B)** *R*^2^ values as a measure for the goodness-of-fit of the linear prediction for the full set of structural features (averaged across different divisions in 5-fold cross-validation). Due to the strong dependencies uncovered in the ensemble of networks, structural features can be reasonably well predicted from activity, with the exception of degree correlations.

Considering the relations between structural features and features of the dynamics, it is a natural question to which degree the structure can be determined by the observed properties of the activity. In order to test if the chosen activity features are sufficient to classify the structure of the underlying graph, we used a linear regression of the values of the structural features based on the values of the activity features across all network realizations. The results are depicted in Figure 6. The scatter plots of the predicted values by means of the optimal regression coefficients vs. the actual values demonstrate the differences between the features (panel **A**). As the standard deviation of the out-degrees affects a variety of activity features, cf. Figure 4, the linear predictions are reasonably accurate. In contrast, the feature IOD, which hardly affects most of the chosen activity variables directly, cannot be predicted as well. As a measure for the goodness-of-fit of the linear regression model, the coefficients of determination, or *R*^2^ values, are plotted in panel **(B)**. They denote the fraction of the variance that is explained by the linear model, and are equal to the squared correlation coefficient between data and linear prediction. As it turns out, only the degree correlation measures IOD and POC cannot be predicted, while other structural features are well approximated even by this fairly elementary predictor.

## 4. Discussion

We studied the effects that variations in the network topology can have on the activity of a population of leaky integrate-and-fire neurons. A better understanding of the effects various local network properties have on the dynamics of the system is an important step toward linking the dynamics of individual neurons to the functional potential of neural populations. Nonetheless, this issue is only beginning to be addressed in the literature. A problem that has only received a limited amount of attention is the interdependence of different aspects of network structure. While the inference of individual connections in networks from spike trains of integrate-and-fire neurons has been studied for example in Shandilya and Timme (2011) and Pernice and Rotter (2013), we are interested in the effects of connectivity statistics on the overall activity. Here, instead of concentrating on a particular feature of a network, we described the dependencies between various aspects in a broad ensemble of simulated networks. Various dependencies between the different features could be found from correlation coefficients across realizations. Although the distributions of features varied strongly and individual quantitative relations can in principle be non-linear and, thus, may potentially not be reflected well by correlations coefficients, we found that non-linear measures like the maximum information coefficient (Reshef et al., 2011) did not suggest additional relationships and thus resulted in qualitatively similar results.

The parameters chosen for our simulations induce a state of irregular firing with low pairwise correlations in random networks through the interplay of supra-threshold external input and inhibition dominance. This activity state arises robustly for strongly simplified as well as more complex neuron models (van Vreeswijk and Sompolinsky, 1996; Brunel, 2000; Kumar et al., 2008). As our results show, it is nonetheless sensitive to the statistics of network connections. This is the case even in a regime well away from any phase boundaries to oscillatory or regular activity states. The similarity of the behavior of different neuron models in a fluctuation driven asynchronous irregular state suggests that relations between features observed in the specific situation analyzed are not sensitive to details of the neuron model or specific parameters. However, for example oscillatory states might be affected in a different manner by variations in the network structure or the neuron model.

Of the various aspects of network structure that are used in the study of networks, some of it in neuroscience, we selected a number characterized by relatively low computational complexity and motivated by experimental observations, as well as their potential effects on dynamics. The influence of some of these network features on the activity has already been analyzed in specific scenarios, while others have received less attention.

The distribution of degrees is one of the standard descriptors used for networks. We observed that the distribution of node degrees has a strong effect on the dynamics. The variance of out-degrees influences the strength of spike train correlations. A large out-degree variance tends to imply strong pairwise and higher-order correlations, as well as a strong power spectrum of the population activity. In contrast, a large variance in the in-degree leads to a broad distribution of firing rates with a high mean, combined with a large fraction of inactive neurons. The influence of broad degree distributions on the emergence of oscillations in recurrent networks was studied in Roxin (2011). In mixed networks of excitatory and inhibitory neurons, a broad in-degree distribution induces oscillations, but not a broad out-degree distribution. An important difference in Roxin (2011) is that only the degree distribution of the excitatory population was varied, while in the current study no difference was made with respect to the type of the neurons. In Zhao et al. (2011) it was noted that the variance of degree distributions is directly related to the occurrence of convergent or divergent motifs. The effects of these motifs, as well as chain motifs, for the tendency of networks to synchronize were analyzed. It was found that only chain and convergent motifs affect this tendency in purely excitatory networks. In contrast, in Hu et al. (2013), a strong effect of divergent and chain motifs on correlations was reported on the basis of a linear approximation, depending on the type of the participating neurons, consistent with the view that divergent motifs correspond to a large amount of shared input within the network, which induces correlations (Shadlen and Newsome, 1998). Contrary to commonly used alternative network models, many of the sampled networks possess strong degree correlations, cf. Figure 1C. In fact, one of the consequences of our sampling procedure is that relatively few networks with uncorrelated degree statistics are found. Nonetheless, the effect of degree correlations is not too striking in our simulations. Positive degree correlations increase the firing rate, but are only weakly related to different dynamical properties. In contrast, in Pernice et al. (2011) degree correlations constrained to the excitatory subnetwork induced strong correlations in a linear neuron model. In Zhao et al. (2011), degree correlations in purely excitatory networks similarly were shown to increase synchrony. Hence, not only overall degree statistics, but also the specificity between different populations are important factors. These results indicate that for the generation of an asynchronous network state by external input combined with recurrent inhibition, the homogeneity of input and output across neurons seems to be an important prerequisite. The experimentally observed broad distribution of firing rates (Barth and Poulet, 2012) can still be realized in these networks, if the non-linear transfer function of neurons is taken into account (Roxin et al., 2011).

The effects of the clustering of neural connections on dynamical properties have been studied in the context of distance dependent connectivity, as in the small world model (Roxin, 2004). However, clustering can arise not only as a consequence of distance dependent connections, but can also be a sign of assemblies defined by a characteristic unrelated to the spatial position of neurons. An increased fraction of reciprocal connections and evidence for clustering in local networks has been reported in Markram (1997); Song et al. (2005), and Perin et al. (2011). Both for the fraction of reciprocal connections and the clustering coefficient, we observed a relation to the average firing rate as well as to the variance of correlations on short timescales, a phenomenon that has been analyzed in Kriener et al. (2009). Reciprocal connections have also been shown to enhance non-normal amplification (Hennequin et al., 2012). If excitatory neurons are organized into clusters, slow rate transients can be observed (Litwin-Kumar and Doiron, 2012). A feature that seems accessible to an exploratory analysis, as the one conducted here, is the frequency of connectivity motifs, for example in three-neuron subnetworks, that has also been studied in experiments (Song et al., 2005). It is, however, difficult to delineate the effects of individual motifs, as the numbers of different motifs especially with larger numbers of connections are strongly correlated among themselves as well as with the clustering within the network. Interestingly, the biological results in Song et al. (2005), Figure 4, suggest an over-representation of divergent, but not of convergent motifs in rat visual cortex, consistent with the assumption of a small in-degree variance, and accordingly high irregularity in neural spike trains. Another interesting feature would be the numbers of specific motifs consisting of excitatory as well as inhibitory connections. An example is the feed-forward inhibition circuit, which was reported to affect the dynamic range of networks (Pouille et al., 2009) as well as to promote the propagation of synchrony (Kremkow et al., 2010) and to enable gating of signals (Vogels and Abbott, 2005). A study about the influence of motifs of this kind will also have to vary the connectivity specific to the type of a neuron.

We also found that activity features are correlated amongst each other. For example, large correlations appear only in networks with high CV. Therefore, although the individual distributions of activity features are broad, not every arbitrary combination of activity features (for instance a regular-synchronous state) can be realized for our present parameter settings.

The activity in individual networks showed that some relations can be attributed directly to single neuron properties. For instance, the property that the activity of neurons with higher rates is more regular in our networks means that networks with higher average firing rates have a smaller average CV. Hence, the relation between in-degree variance and CV ultimately arises from the setting of supra-threshold external input combined with recurrent inhibition. Previous studies have already related the activity of individual neurons to their input statistics (Hamaguchi et al., 2011). There, CV-rate curves of single neurons where attributed to changes in the external input and used to infer global parameters of the local network. Our results suggest that the variations across neurons under stationary conditions could be used alternatively. The consideration of additional features of the activity distribution can potentially be used to gather information about supplementary structural characteristics. However, one has to keep in mind that the effects of topology can also depend strongly on dynamical properties of the single neurons as well as on the global activity regime.

For the first time, to our knowledge, our study assesses the presence of higher-order correlations (HOC) in large recurrent networks of neurons. In retinal recordings of about 100 neurons interaction parameters up until the order five in a maximum entropy model were used to describe activity patterns (Ganmor et al., 2011). The studies (Macke et al., 2011; Yu et al., 2011) found evidence that HOC are shaped by the amount of common input that neurons receive. Using a novel statistical hypothesis test based on the cumulants of the population spike count (Staude et al., 2010b), we found that in networks of 12,500 cells, the highest significant order of HOC, as measured by the minimal order of joint cumulants between single spike trains needed to explain activity statistics, ranges up to about 500, with a mean of 81 (cf. Figure 3A and Table 4). The relation between the maximum-entropy model and the cumulant based concepts of HOC is discussed in Staude et al. (2010a).

Apart from the network characteristics considered here, there are many potentially more complex topologies to be analyzed. We have not considered the modularity of our networks (Leicht and Newman, 2008). The presence of hierarchical connectivity can also affect the dynamics (Müller-Linow et al., 2008; Jarvis et al., 2010; Kaiser and Hilgetag, 2010). As our networks are not embedded in space, no distance between neurons can be defined, and consequently effects of the distance dependence of neural connections which can lead to spatial patterns like propagating waves (Roxin, 2004; Voges and Perrinet, 2009, 2012), are not taken into account. Especially the connectivity between excitatory and inhibitory connections was not changed independently so that further effects related to this feature remain to be explored.

### Conflict of interest statement

The authors declare that the research was conducted in the absence of any commercial or financial relationships that could be construed as a potential conflict of interest.
